# The Cellular and Subcellular Organization of the Glucosinolate–Myrosinase System against Herbivores and Pathogens

**DOI:** 10.3390/ijms23031577

**Published:** 2022-01-29

**Authors:** Qiaoqiao Lv, Xifeng Li, Baofang Fan, Cheng Zhu, Zhixiang Chen

**Affiliations:** 1 College of Life Sciences, China Jiliang University, Hangzhou 310018, China; s20090710039@cjlu.edu.cn (Q.L.); 19a0902115@cjlu.edu.cn (X.L.); 2 Purdue Center for Plant Biology, Department of Botany and Plant Pathology, Purdue University, West Lafayette, IN 47907-2054, USA; bfan@purdue.edu

**Keywords:** glucosinolates, plant chemical defense, myrosinases, myrosin cells, ER body, mustard bomb

## Abstract

Glucosinolates are an important class of secondary metabolites in *Brassicales* plants with a critical role in chemical defense. Glucosinolates are chemically inactive but can be hydrolyzed by myrosinases to produce a range of chemically active compounds toxic to herbivores and pathogens, thereby constituting the glucosinolate–myrosinase defense system or the mustard oil bomb. During the evolution, *Brassicales* plants have developed not only complex biosynthetic pathways for production of a large number of glucosinolate structures but also different classes of myrosinases that differ in catalytic mechanisms and substrate specificity. Studies over the past several decades have made important progress in the understanding of the cellular and subcellular organization of the glucosinolate–myrosinase system for rapid and timely detonation of the mustard oil bomb upon tissue damage after herbivore feeding and pathogen infection. Progress has also been made in understanding the mechanisms that herbivores and pathogens have evolved to counter the mustard oil bomb. In this review, we summarize our current understanding of the function and organization of the glucosinolate–myrosinase system in *Brassicales* plants and discuss both the progresses and future challenges in addressing this complex defense system as an excellent model for analyzing plant chemical defense.

## 1. Introduction

Plants have a variety of inducible and constitutive mechanisms including chemical defenses to protect themselves against attack by pathogens and herbivores. Plant chemical defenses are associated with a vast array of antimicrobial and antiherbivory secondary metabolites, predominantly terpenoids, phenolics, and N-containing compounds (including alkaloids, cyanogenic glycosides, glucosinolates, and benzoxazinoids) [1,2,3]. In addition, some plants use fatty acid derivatives, amino acid, and even peptides as defense chemicals [4,5,6]. Production of plant defense chemicals can be inducible or constitutive. For examples, some antimicrobial compounds are rapidly synthesized at infection sites in response to microbial pathogens and are usually referred to as phytoalexins [7]. Phytoalexins are chemically diverse and broad-spectrum inhibitors of microbial organisms. By contrast, phytoanticipins are preformed antimicrobial compounds that are present in plants before attack by microorganisms or infection [7]. Many of these antimicrobial chemicals are also effective against other types of organisms including herbivores and, therefore, the classification of defense chemicals can be broadened to include those that respond to insect pests. Plants also generate volatile organic compounds with major roles in plant communication with the surrounding organisms including microorganisms, pollinators, herbivores, and other natural enemies [8,9].

**Figure 1:**
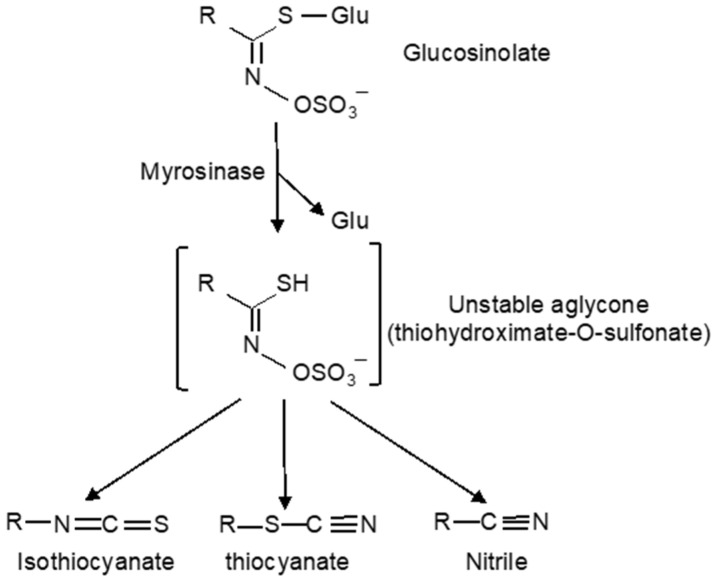
The glucosinolate–myrosinase defense system. Glucosinolates are hydrolyzed by myrosinases upon tissue damage to generate unstable aglycones, which can rearrange to produce chemically active isothiocyanates, thiocyanates, and nitriles.

Glucosinolates, which are widely distributed in the order *Brassicales*, are one of the most extensively analyzed classes of defense chemicals in plants [10,11] (Figure 1). Upon tissue damage by pests, glucosinolates are hydrolyzed by thioglucoside glucohydrolases (TGGs) called myrosinases into unstable thiohydroximate- O-sulfonates, which can rearrange to form a range of hydrolytic products including isothiocyanates, nitriles, and other by-products toxic to herbivores, pathogens, and other organisms [12] (Figure 1). This glucosinolate–myrosinase defense system is also known as “mustard oil bomb” [13,14]. Over the past two decades or so, important progress has been made in the analysis of the structural diversity of glucosinolates and their metabolites. More importantly, a great deal has been learned from the extensive research in the model plant *Arabidopsis* on the cellular and subcellular organization of the mustard oil bomb required for its rapid and timely activation upon tissue damage. Important information has also been gained on how some herbivores and pathogens disarm the mustard oil bomb or even exploit it for defense against predators. In the review, we discuss what we currently know, what questions remain, and how a better knowledge about the glucosinolate–myrosinase system, particularly about the diversity, function, spatial organization, and evolution of the components of the mustard oil bomb, can help in understanding the molecular and cellular basis of chemical defense in plants.

## 2. The Glucosinolate–Myrosinase Chemical Defense System

Glucosinolates are a well-defined class of metabolites produced by cabbages, mustards, and related plants in the *Brassicales* order [10,11]. After hydrolysis, all glucosinolates have the potential to form isothiocyanates, the pungent constituent in domestic mustard with a sharp taste, and, therefore, are historically known as mustard oils [10,11]. Glucosinolates are nitrogen-containing, sulfur-rich, amino acid-derived metabolites with a β-d-glucopyranose unit linked through a sulfur atom to an *N*-hydroxyimino sulfate ester [15,16] (Figure 1). Besides the β-thioglucose and thiohydroximate-O-sulfonate compartments, glucosinolates contain a variable aglycone side chain derived from an α-amino acid, which can be either aliphatic, indole, or aromatic [15,16]. Glucosinolates are synthesized from amino acids (alanine, leucine, isoleucine, methionine, phenylalanine, tryptophan, tyrosin, and valin) in three phases: chain elongation of precursor amino acids (especially methionine), formation of the glucosinolate core structure, and further side-chain modification. Most of the enzymes involved in the core structure biosynthesis have been identified and cloned in *Arabidopsis* [10,11,17], and it is possible now to engineer the production of glucosinolates in non-cruciferous organisms by transferring an entire glucosinolate biosynthetic pathway [18,19,20,21,22,23].

Glucosinolates are usually stored in the vacuole in plant cells. Upon tissue damage after pest feeding or other mechanical disruption, glucosinolates are hydrolyzed by myrosinases into unstable thiohydroximate- O-sulfonates, which rearrange to form different hydrolytic products such as isothiocyanates, nitriles, and other by-products depending on the side chains of glucosinolates, the reaction conditions including iron and pH, and myrosinase interacting proteins [12,24] (Figure 1). The toxic hydrolysis products of glucosinolates have a variety of biological activities in insect–plant interactions, primarily as defense compounds against herbivores and pathogens. Upon ingestion after consumption of cruciferous vegetables by human, glucosinolates break down to isothiocyanates and indoles, which have health benefits such as decreasing inflammation and lowering the risk of cancer [25,26,27,28].

There are two types of myrosinases: typical (classical) and atypical myrosinases. Classical myrosinase proteins fold into an (*β*/*α*)_8_ barrel structure and involve a glutamate (E) residue in the active site to initiate the release of an aglycone (thiohydroximate-O-sulfonate) by nucleophilic attack and form a glucosyl–enzyme intermediate [29]. Another glutamine (Q) residue is also required for the subsequent hydrolysis of the glucosyl–enzyme intermediate with assistance from ascorbate, which acts as a cofactor and proton donor to promote the release of bound glucose [29,30,31]. Classical myrosinases only use glucosinolates as the substrates [12,32]. On the other hand, atypical myrosinases have two catalytic glutamate residues (EE) as acid/base catalyst in the active site but do not require ascorbate as a cofactor [33]. Atypical myrosinases also contain additional basic amino acid residues at specific positions for glucosinolate binding [30,31,33,34]. Unlike classical myrosinases, atypical myrosinases can both indole glucosinolates and O-glucosides as substrates [33,35].

Both classical and atypical myrosinases belong to glycoside hydrolase family 1, which, in *Arabidopsis*, is composed of 47 BETA-GLUCOSIDASE (BGLU) genes and a BGLU-like gene, AFR2 [36]. There are six BGLU genes (*BGLU34-BGLU39*) encoding classical myrosinases (*TGG1*-*TGG6*) [36]. *TGG1* and *TGG2* are primarily expressed in leaves [37,38,39,40,41] and flowers [37,42], while *TGG4* and *TGG5* are specifically expressed in roots [43]. *TGG3* and *TGG6* are non-functional pseudogenes [38,44]. Atypical myrosinases include PEN2/BGLU26 and PYK10/BGLU23, which have been extensively analyzed in defense against pathogens and herbivores [33,35,45,46]. Both the two EE catalytic residues at the active site and additional basic residues in the substrate-binding pocket identified in PEN2/BGLU26 and PYK10/BGLU23 are conserved among 16 *Arabidopsis* BGLU genes (BGLU18-33), indicating that other members of this BGLU subfamily may also have myrosinase activities [33]. In *Arabidopsis* mature leaves, the classical TGG1 and TGG2 myrosinases and glucosinolates accumulate in two different types of cells [34]. This dual-cell type of chemical defense is activated upon tissue damage, which allows myrosinases gain access to glucosinolates to produce the toxic compounds. In *Arabidopsis* seedlings, on the other hand, high levels of atypical myrosinases PYK10/BGLU23 and related β-glucosidase accumulate in the endoplasmic reticulum (ER)-derived organelles called ER bodies and can obtain access to the glucosinolates stored in the vacuole in the same cell upon tissue damage, enabling a single-cell chemical defense system [47,48].

## 3. The Dual-Cell Type of Mustard Oil Bombs

**Figure 2:**
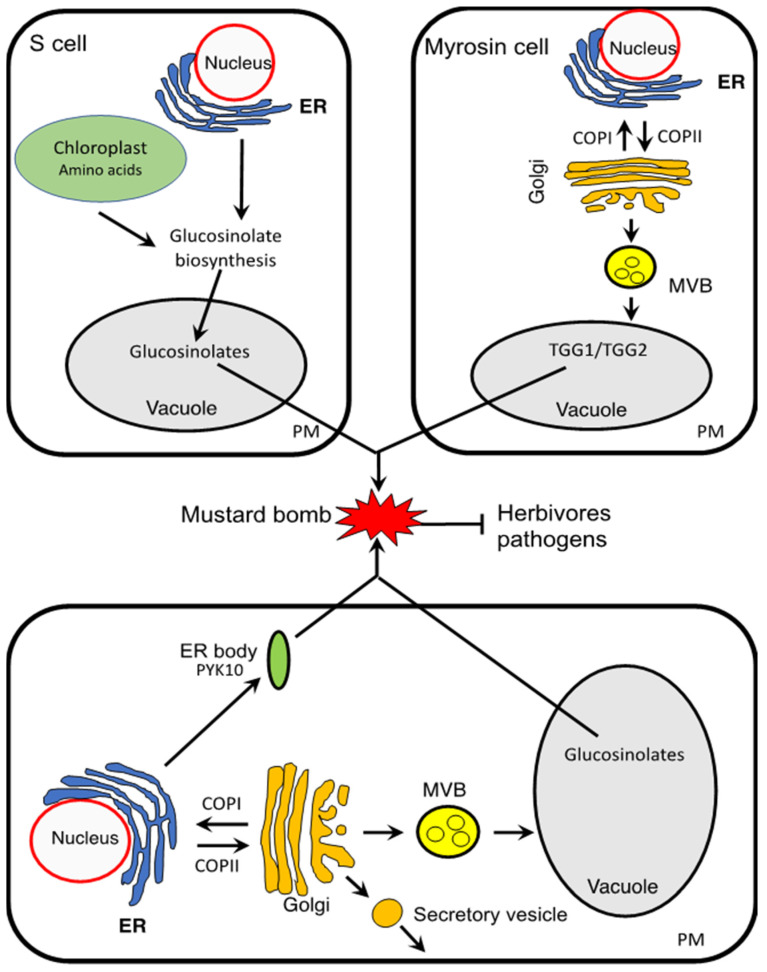
Two types of the mustard oil bomb. In the dual-cell type of the mustard bomb, glucosinolates are stored in the S cells, whereas classical myrosinases such as TGG1 and TGG2 from *Arabidopsis* accumulate in the vacuole of myrosin cells. In the single-cell type mustard oil bomb, atypical myrosinases such as PYK10 and glucosinolates accumulate in the ER bodies and vacuole of the same cell. Upon tissue damage, myrosinases obtain access to glucosinolates to detonate the mustard oil bomb by generating chemically active compounds toxic to herbivores and pathogens. ER, endoplasmic reticulum; PM plasma membrane; MVB, multivesicular body.

In the dual-cell type of mustard oil bombs, glucosinolates and myrosinases are localized in different cells to maintain their stability (Figure 2). Glucosinolates are present in organs throughout the plant from roots to flowers. However, glucosinolates are particularly high in a special type of cells called S-cells based on their high sulfur content [49]. In *Arabidopsis*, S-cells exist as groups between the endoderms and the phloem cells of each vascular bundle [49]. On the other hand, classical myrosinases localized to protein-accumulating idioblasts called myrosin cells [39,50,51] (Figure 2). In *Arabidopsis*, *TGG1* and *TGG2* are expressed specifically in myrosin cells along leaf veins and stomatal guard cells [37,38,39,40,41]. TGG1 and TGG2 proteins are abundant in aerial organs of *Arabidopsis* plants but their levels in myrosin cells are usually much higher than in guard cells [40,52]. Both glucosinolates and myrosinases are stored in the vacuole of their respective S- and myrosin cells [34].

Other studies in different plant species have, however, presented a more complex or even contradictory picture about the cellular compartmentation of the dual-cell mustard oil bomb. In *Brassica juncea* seedlings, it was found that myrosinases co-localize with glucosinolates in aleurone-type cells [53]. Even in *Arabidopsis*, both myrosinases and GLSs are present in suspension cells [54]. Additionally, guard cells contain not only myrosinases but also glucosinolates, as confirmed by metabolomics data [55,56]. GLS metabolism is altered in guard cells upon treatment with CO_2_ [55], and ABA [56] and plays a role in stomatal movement based on the analysis of the effect of ABA on stomatal movement of the *Arabidopsis* myrosinase mutant *tgg1* [57] and other genetic mutants [58,59]. Furthermore, stomatal closure was induced by pharmacological treatments with different glucosinolate hydrolysis products [60,61]. Given the expression of TGG genes in guard cells, it is very likely that the classical myrosinases are involved in the glucosinolate metabolism in guard cells. Therefore, classical myrosinases can also be present in the same cells as glucosinolates, and there are additional mechanisms such as spatial separation at the subcellular levels and tight control of myrosinase activities that play a role in the stability of the mustard oil bombs.

## 4. The Single-Cell Type of Mustard Oil Bomb

The single-cell type of mustard oil bomb involved ER-derived organelles (ER bodies) that accumulate atypical myrosinases and the vacuole that stores glucosinolates in the same cell (Figure 2). Like glucosinolates, ER bodies are present in the *Brassicales* order, including *Arabidopsis* [47]. ER bodies have a rod shape, about 1 μm in diameter and 10 μm in length, and were originally reported from transgenic *Arabidopsis* plants expressing ER-targeted GFP [62,63]. Under electron microscopy, ER bodies have a single membrane covered by ribosomes and are continuous to the ER network [63]. Based on tissue specificity, ER bodies have been classified into two types: (i) constitutive ER bodies present in the epidermal cells of the cotyledons, hypocotyls, and roots and (ii) wound/jasmonic acid (JA)-inducible ER bodies in the rosette leaves of *Arabidopsis*. More recently, it has been reported that there is a third type of ER bodies known as leaf ER bodies that is constitutively present in specific cells of rosette leaves (marginal cells, epidermal cells covering the midrib, and giant pavement cells) [64]. PYK10/BGLU23, an atypical myrosinase with a KDEL ER retention signal at its C terminus, is the major protein component of the constitutive ER bodies in *Arabidopsis* [65]. Constitutive ER bodies in *Arabidopsis* also accumulate at the membrane two integral membrane proteins with a metal ion transporter activity, MEMBRANE OF ER BODY1 (MEB1) and MEB2 [66]. Wound-inducible ER bodies, on the other hand, accumulate primarily BGLU18 [67], another KDEL-tailed β-glucosidase family, whereas leaf ER bodies accumulate both PYK10/BGLU23 and BGLU18 [64].

There are eight genes encoding KDEL-tailed BGLU proteins (BGLU18 to 25) in *Arabidopsis*. Biochemical analysis indicates that these BGLU proteins in the ER bodies have a myrosinase activity that hydrolyzes glucosinolates to generate chemically reactive products toxic to pathogens and herbivores [33]. Apparently, in the seedlings, the atypical myrosinases and glucosinolates are stored in ER bodies and vacuole, respectively, in the same cells and gain access to each other upon tissue damage to produce toxic products, thereby constituting a mustard bomb that operates through a single-cell mechanism [48] (Figure 2). Importantly, there is a striking co-expression pattern among genes associated with the ER body, glucosinolate biosynthesis, and metabolism, indicating strong coordination among these processes [33]. The role of the single-cell type of mustard oil bomb in plant chemical defense is supported by the finding that *Arabidopsis* mutants deficient in the ER body formation are hypersusceptible to herbivores such as woodlice and the chewing insect *Spodoptera exigua* [48,68]. In the ER body-deficient *Arabidopsis* mutants, there is also overgrowth of the beneficial fungus *Piriformospora indica* without beneficial effects on the plants [69]. Thus, ER body formation is important in plant defense that enables an appropriate level of fungal colonization to establish a mutualistic interaction between the symbiotic partners [69]. The ER body may also play a role in plant responses to abiotic stresses, including drought and metal ion toxicity [66,70].

Genetic analysis in *Arabidopsis* has identified two genes, *NAI1* and *NAI2*, with a critical role in the ER body formation in *Arabidopsis* [71,72]. *NAI1* encodes a bHLH-type transcription factor and functions as a master regulator of the ER body formation by regulating the expression of genes associated with ER bodies including PYK10/BGLU23, *NAI2*, MEB1, and MEB2 [71]. *NAI2* encodes an ER body component that is required for the constitutive ER body formation in *Arabidopsis* [72]. In the *nai2* mutants, PYK10/BGLU23, MEB1, and MEB2 proteins are diffused throughout the ER and the levels of PYK10 are reduced, indicating that the formation of the ER bodies promotes accumulation of PYK10 [72]. NAI2 forms complexes with MEB1 and MEB2 and, therefore, may function as receptors or adaptor for the recruitment and organization of these ER body cargo proteins [66]. In *Arabidopsis*, NAI2 has a close homolog, TONSOKU (TSK)-ASSOCIATED PROTEIN1 (TSA1), which is required for wound/JA-induced ER body formation [73]. Like ER bodies and glucosinolates, homologs of NAI2 and TSA1 are found only in plants in the *Brassicaceae* order, suggesting that NAI2 and its homologs have evolved specifically for the formation of the ER bodies [72].

## 5. Development and Evolutionary Origin of Myrosin Cells

**Figure 3:**
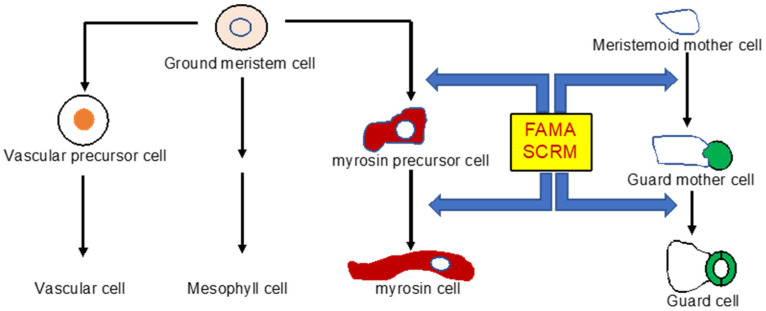
Development of myrosin cells in *Arabidopsis*. Myrosin cells are differentiated from ground meristem cells, which are also the mother cells for vascular cells and mesophyll cells. The FAMA and SCRM transcription factors function as master regulators of both myrosin and guard cell differentiation.

Myrosin cells are found along veins in *Arabidopsis* and other *Brassicales* plants [74,75,76]. Based on the close proximity of myrosin cells to vascular precursor cells and phloem cells [50,75,76], myrosin cells could belong to a vascular cell lineage and differentiate from vascular and vascular precursor cells. More recent studies, however, have provided evidence against this hypothesis. Instead, these studies have shown that myrosin cells are differentiated directly from ground meristem cells [52,75,77] (Figure 3). Ground meristem cells, located in the inner tissues of leaf primordia, are also the mother cells for vascular precursor cells and mesophyll cells [78,79] (Figure 3). Ground meristem cells can first differentiate into isodiametric small myrosin precursor cells, which mature into the large and irregular idioblast myrosin cells [52,75,77]. Therefore, myrosin cells are developmentally independent of the vasculature in *Arabidopsis* leaves [34].

A number of basic helix-loop-helix (bHLH) transcription factors including FAMA, SCREAM (SCRM), and SCRM2 function as master regulators of myrosin cell differentiation [52,77] (Figure 3). FAMA expression starts in isodiametric small cells, and its mutation abolishes myrosin cell differentiation and accumulation of TGG1 and TGG2 [52,77]. Overexpression of FAMA, on the other hand, leads to production of a large number of myrosin cells [52]. SCRM and SCRM2 interact with FAMA and play a redundantly essential role in myrosin cell development [52]. Importantly, these three transcription factors are also master regulators of guard cell differentiation [80,81] (Figure 3). Thus, myrosin cell development is regulated by transcriptional network similar to that in guard cell development. FAMA–SCRM/2 heterodimers, as common master regulators of the development of both myrosin cells and guard cells, also provide strong evidence that these two types of cells are evolutionarily related. Guard cells are present in all plants including moss and hornwort [82]. In contrast, TGG-accumulating myrosin cells appeared much later only in *Brassicales* plants [83]. It has been proposed that an ancestral *Brassicales* plant first accumulated glucosinolates after TGG1 or a similar myrosinase became connected with FAMA-mediated transcriptional cascade in guard cells [34]. The ancestral *Brassicales* plants accumulated glucosinolates in stomatal guard cells and produced isothiocyanates, which could be transported to the extracellular region by proteins such as PEN3 for defense against invading pathogens [84,85]. The ancestral *Brassicales* plants subsequently acquired myrosin cells along leaf veins when FAMA expression began in ground meristem cells of inner leaf tissues during evolution.

## 6. Evolutionary Origin of ER Bodies

As sessile organisms, plant cells highly regulate their endomembrane system including the ER [86]. In addition, plants produce several types of functionally specialized ER-derived vesicles [87,88,89,90,91,92]. Unlike COPII vesicles in the classical secretory pathway, these specialized ER-derived vesicles carry specific proteins but do not travel through the well-characterized ER-to-Golgi transport pathway. Some of these ER-derived vesicles such as protein bodies accumulate storage proteins and can exist as independent storage organelles or traffic specific storage proteins directly from the ER to the storage vacuole in a Golgi-independent manner [88,93]. Other specialized ER-derived vesicles such as ER bodies accumulate proteases and hydrolytic enzymes with roles in plant growth, development and stress responses [87]. While all plants can form protein bodies, ER bodies are formed only in *Brassicaceae* plants [72]. An important evolutionary question about ER bodies is where they arose. Did they originate de novo in *Brassicaceae* plants or evolve from preexisting ER structures? We have recently provided important insights into the evolutionary origin of the ER bodies based on the analysis of three closely related NAI2-interacting proteins (NAIP1, 2, and 3) from *Arabidopsis* [94]. The three NAIP all contain a C-terminal region homologous to the protein-binding harmonin homology domain (HHD) that interacts with NAI2. The three proteins also contain a similar N-terminal coiled-coil (CC) domain. The middle regions of NAIPs are highly divergent but all contain multiple threonine/serine-proline phosphorylation sites by proline-directed protein kinases such as mitogen-activated protein kinases and cyclin-dependent protein kinases [95]. Thus, the NAIP proteins contain multiple protein-interacting motifs and are potentially subjected to regulation by protein phosphorylation. There is no homolog of NAIPs in the archaea, eubacteria, fungi, or animals. NAIP homologs are found in the phylum of Apicomplexa in the large clade of parasitic alveolate in protista [94]. Importantly, there are NAIP homologs in all plants including the unicellular green alga *Chlamydomonas reinhardtii*, the moss *Physcomitrella patens*, the fern *Selaginella moellendorffii*, and in both angiosperms and gymnosperms. Thus, NAIP proteins have originated in early eukaryotes and are present in all land plants, typically as a small family of three to four members [94].

**Figure 4:**
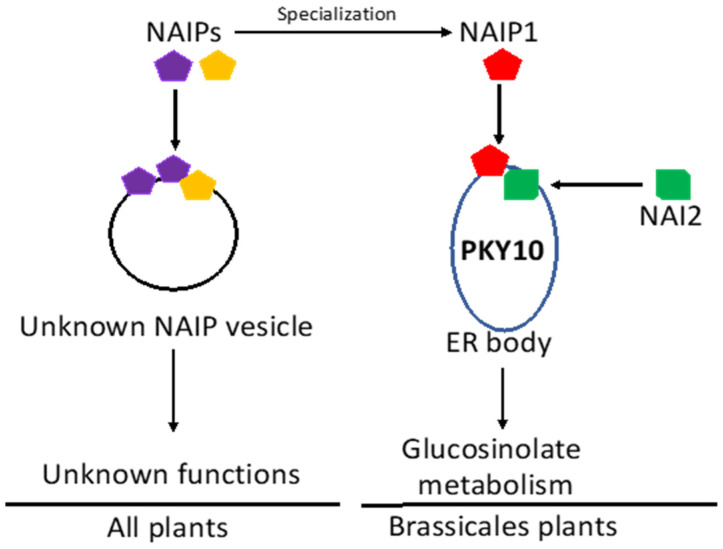
A proposed model for the evolutionary origin of the ER bodies. NAIP proteins are critical components for a family of ER-derived vesicles that are present in all plants. NAIP1 became specialized to be specifically associated with ER bodies through interaction with NAI2. ER bodies accumulate atypical myrosinases such as PYK10 and play a critical role in glucosinolate metabolism in defense against herbivores and pathogens.

Genetic analysis showed that constitutive ER body formation is almost completely abolished in the *naip1*/*naip2*/*naip3* triple mutant, as in the *nai2* mutant, but is normal in the *naip* single and *naip1*/*naip2* double mutants [94]. Thus, NAIPs play a critical and redundant role in the ER body formation [94]. Studies using NAIP-GFP fusions further revealed that NAIP1 formed punctate structures in a tissue-specific pattern identical to those of known ER body markers and in an NAI2-dependent manner, indicating that NAIP1 is specifically associated with the ER bodies [94]. On the other hand, NAIP2- and NAIP3-GFP are associated not only with the ER bodies but also with other unknown vesicular structures whose formation is ubiquitous and NAI2 independent. Based on these findings, we have proposed that the NAI2/TSA1-containing ER bodies in the *Brassicales* may have evolved from a family of preexisting NAIP-containing ER-derived structures widely present not only in plants [94] (Figure 4). In *Arabidopsis*, NAIP1 has evolved to be functionally highly specialized for ER body formation, whereas NAIP2 and NAIP3 are less specialized and function as components of not only the ER bodies but also other ER-derived structures that can be formed in a wider range of plant tissues [94] (Figure 4). It remains to be determined about the biochemical and functional nature of the other NAIP-containing vesicles, which the ER bodies are related to and likely evolved from. What cargo proteins do they carry? Do they also have roles in plant defense?

## 7. Countering and Exploiting the Mustard Oil Bomb by Pests and Pathogens

**Figure 5:**
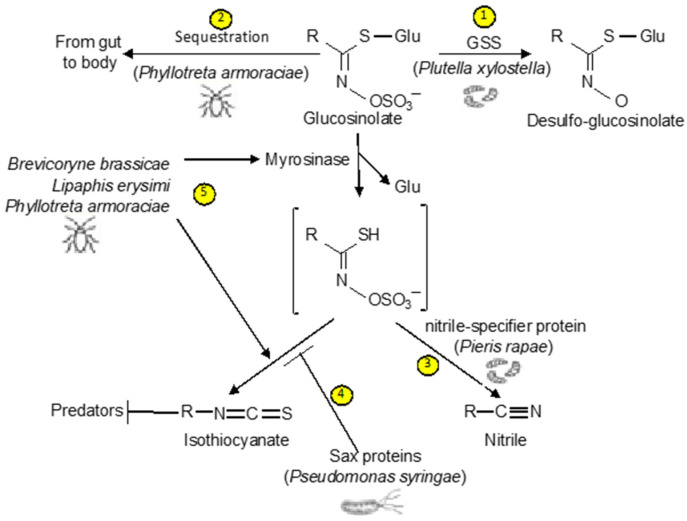
Mechanisms by pests and pathogens to counter or exploit the glucosinolate–myrosinase system. These mechanisms include inactivation of glucosinolates by glucosinolate sulfatase (GSS) (1) or sequester glucosinolates from the gut to the body (2). Other pests rely on nitrile-specifier proteins to redirect the glucosinolate hydrolysis from isothiocyanates, which are highly toxic, toward nitriles, which are less toxic (3). Bacterial Pseudomonas pathogens also use Sax proteins to reduce isothiocyanate formation (4). Some adapted specialist insects sequester glucosinolates and convert these glucosinolates to toxic products by using their own myrosinase for defense against predators (5).

The glucosinolate–myrosinase system protects *Brassicales* plants against herbivores and pathogens with toxic hydrolytic products of glucosinolates. On the other hand, many herbivores and pathogens have also evolved different mechanisms that counter the mustard oil bomb. These mechanisms include modification of glucosinolates to prevent formation of toxic hydrolytic products and metabolic diversion that prevent formation of highly toxic to less toxic metabolites. The diamondback moth (*Plutella xylostella*), a crucifer specialist insect, produces glucosinolate sulfatase as a gut content that catalyzes the hydrolysis of glucosinolates to produce desulfo-glucosinolates, which are not myrosinase substrates [14] (Figure 5). Importantly, the glucosinolate sulfatase acts on all major classes of glucosinolates, thus effectively disarming the mustard oil bomb and enabling diamondback moths to use a broad range of cruciferous host plants [14]. Another specialist insect herbivore, *Pieris rapae*, one of the most abundant butterflies and pests in Northern and Central Europe and in North America, uses a different counter-adaptation mechanism that enables them to feed on *Brassicales* plants without severe negative effects [96] (Figure 5). There is a larval gut protein from *P. rapae* that redirects the glucosinolate hydrolysis toward nitrile formation instead of isothiocyanates [96]. This protein, designated the nitrile-specifier protein, has no hydrolytic activity on glucosinolates by itself and is unrelated to any functionally characterized protein [96]. Isothiocyanates are highly reactive compounds toxic to a broad range of organisms (including *P. rapae*, whereas nitriles are generally less toxic than isothiocyanates, and larvae of *P. rapae* excrete nitriles in the feces with or without further metabolism) [97,98]. How the nitrile-specifier protein alters the metabolic products of glucosinolates is still unknown. The protein itself does not have hydrolytic activity on glucosinolates but may serve as a cofactor of plant myrosinase to alters the direction of glucosinolate hydrolysis [96].

Other herbivores develop other mechanisms such as sequestering glucosinolates and inhibition of plant myrosinases to counter the mustard oil bomb. The specialist flea beetle, *Phyllotreta armoraciae*, is capable of absorbing from the gut high levels of glucosinolates in the body and can thus at least partially avoid plant myrosinase activity [99] (Figure 5). Interestingly, feeding experiments with the myrosinase-deficient *Arabidopsis tgg1*/*tgg2* mutant and wild type plants indicate that plant myrosinases reduced the glucosinolate sequestration rate by the insect in adult beetles [99]. Further analysis revealed that *P. armoraciae* can inactivate plant myrosinases in the gut to both reduce glucosinolate hydrolysis and promote their sequestering [99]. These findings indicate that adaptations of *P. armoraciae* to their brassicaceous host plants involve both the ability to tolerate plant myrosinase activity and a fast glucosinolate uptake mechanism.

Microbial pathogens also have mechanisms to counter the mustard oil bomb. Sulforaphane (4-methylsulfinylbutyl isothiocyanate), a natural product derived from aliphatic glucosinolates, inhibits growth of non-host Pseudomonas bacteria in *Arabidopsis* plants [100]. The survival in *Arabidopsis* extracts genes (saxCAB/F/D/G) identified in *Pseudomonas* species virulent on *Arabidopsis* are required to overwhelm isothiocyanate-based defenses and facilitate a disease outcome, especially in the young leaves critical for plant survival [100] (Figure 5). As a result, a virulent *P. syringar DC3000* pathogen lacking SaxA/B/F/D/G genes could not grow in young *Arabidopsis* leaves but could grow in young leaves of *Arabidopsis myb28 myb29* mutants, which did not produce aliphatic glucosinolates [100]. The accumulated amounts of the major glucosinolates were unchanged in plants overexpressing SaxA, suggesting that SaxA inhibits aliphatic isothiocyanate production after glucosinolate breakdown [100]. These combined results indicate that aliphatic isothiocyanates are effective not only in limiting damage by herbivores but also in defense against microbial bacterial pathogens, and, therefore, there is also an arms race between *Brassicales* plants and their pathogens.

Some adapted herbivore specialists have also evolved mechanisms that exploit glucosinolates produced by host plants for defense against their predators or for interspecific communication. In two aphid species, *Brevicoryne brassicae* and *Lipaphis erysimi*, these specialist insects sequester glucosinolates and convert these glucosinolates to toxic products by using their own myrosinase, encoded in the aphid genome [101,102,103,104] (Figure 5). It has been shown that the higher level of glucosinolates in *B. brassicae* had a significant negative impact on survival of *Adalia bipunctata*, a ladybird predator [104]. Likewise, flea beetles of the genus Phyllotreta possess the glucosinolate–myrosinase defense system that consists of sequestered glucosinolates from plant hosts an insect myrosinases capable of converting the non-toxic glucosinolates to deterrent isothiocyanates [105] (Figure 5). A comparison of two different stages of the horseradish flea beetle *P. armoraciae* showed that the larvae contained 1.5-fold less glucosinolates but 43.4-fold higher myrosinase activity than the pupae [106]. Importantly, while larvae produced high amounts of toxic isothiocyanates when they were attacked by the generalist predator *Harmonia axyridis* and deterred the predator and survived on attack, the pupae did not produce high levels of the toxic products of glucosinolates and were killed [106]. The glucosinolate–myrosinase system has also been exploited by microbial pathogens to counter plant defense. For example, ER bodies are induced by the bacterial pathogen *Pseudomonas syringae* in a manner dependent on the bacterial toxin coronatine but play a negative role in immunity against the bacterial pathogen [68]. Thus, the bacterial pathogen exploits the ER bodies as a counter-defense mechanism to promote virulence.

## 8. Summary and Prospect

Glucosinolates or mustard oil were first reported more than 60 years ago [107,108,109]. A major area of research on glucosinolates has been their identification, chemical diversity, and metabolism in different *Brassicales* plant species [12,16]. With the development of the *Arabidopsis* model system, isolation and functional analysis of genes responsible for the biosynthesis and metabolism of glucosinolates have been greatly accelerated. Great progress has also been made in the discovery of multiple classes of myrosinases and the distinct cellular and subcellular organization of the mustard oil bomb. The molecular and evolutionary events that lead to the cellular and subcellular organization of the mustard oil bomb have also emerged from recent molecular and genetic analysis in *Arabidopsis*. In addition, novel mechanisms evolved in herbivores and pathogens to overcome the mustard oil bomb have also been discovered, which underscore the critical role of the glucosinolate–myrosinase system in plant chemical defense.

Even though a great deal has been learnt about the glucosinolate–myrosinase system, many important questions remain elusive about the complex defense system. With the increased sensitivity of metabolic profiling, the number of glucosinolates in different *Brassicales* plants continues to increase, raising new questions about the dynamic evolution of the important group of secondary metabolites with implications not only for plant interactions with other organisms but also for the health benefit of human consumption of cruciferous vegetables. Even though there is clear and strong evidence for the spatial separation of glucosinolates and classical myrosinases for the dual cell-type of the mustard oil bomb, there are tissues and cells where the two components of the mustard oil co-exist, and it remains to be determined how the stability of glucosinolates is maintained in the presenc of myrosinases. A large number of myrosinase-associated proteins have been identified but our understanding of their roles in the regulation of the activity, substrate specificity, and other properties of myrosinases including the products that are generated from the hydrolysis of glucosinolates is very limited. Even though strong evidence for the evolutionary origin of both the myrosin cells and ER bodies have emerged, further research will be necessary to identify additional factors to fully establish the molecular and cellular machineries or network required for the differentiation of myrosin cells and ER body biogenesis. This information will be necessary to engineer the lucosinolate–myrosinase system in non-cruciferous plants. Finally, even though a number of mechanisms by which herbivores and pathogens overcome the glucosinolate–myrosinase defnese system have been reported, it is unclear whether there is a co-evolutionary history of arms race of defense and counterdefense between plants and the pests. This knowledge has important ecological and agricultural implications and can also provide useful information on the strategies and targets for pest management.

## Data Availability

Not applicable.
